# Expansion of US wood pellet industry points to positive trends but the need for continued monitoring

**DOI:** 10.1038/s41598-020-75403-z

**Published:** 2020-10-29

**Authors:** Francisco X. Aguilar, Ashkan Mirzaee, Ronald G. McGarvey, Stephen R. Shifley, Dallas Burtraw

**Affiliations:** 1 Department of Forest Economics, Swedish University of Agricultural Sciences, 90 183 Umeå, Sweden; 2 Department of Industrial and Manufacturing Systems Engineering, University of Missouri, Columbia, MO 65211 USA; 3 Department of Industrial and Manufacturing Systems Engineering and Harry S Truman School of Public Affairs, University of Missouri, Columbia, MO 65211 USA; 4 School of Natural Resources, University of Missouri, Columbia, MO 65211 USA; 5 Resources for the Future, Washington, DC 20036 USA

**Keywords:** Environmental social sciences, Energy and society, Environmental impact, Sustainability, Forestry

## Abstract

Implementation of the European Union Renewable Energy Directive has triggered exponential growth in trading of pelletized wood fibers. Over 18 million tons of wood pellets were traded by EU member countries in 2018 of which a third were imported from the US. Concerns exist about negative impacts on US forests but systematic assessments are currently lacking. We assessed variability in fundamental attributes for timberland structure and carbon stocks within 123 procurement landscapes of wood pellet mills derived from over 38 thousand forest inventory plots in the eastern US from 2005 to 2017. We found more carbon stocks in live trees, but a fewer number of standing-dead trees, associated with the annual operation of large-scale wood pellet mills. In the US coastal southeast—where US pellet exports to the EU originate—there were fewer live and growing-stock trees and less carbon in soils with every year of milling operation than in the rest of the eastern US—which supplies the domestic market. Greater overlap of mills’ procurement areas exhibited discernible increments across selected carbon stocks. These trends likely reflect more intensive land management practices. Localized forest impacts associated with the wood pellet industry should continue to be monitored.

## Introduction

The expansion of renewable energy production offers an alternative to reduce net greenhouse gas emissions while contributing to socio-economic and environmental objectives^1^. The European Union’s Renewable Energy Directives—RED and RED II, henceforth collectively referred to as EU RED—set ambitious goals for the substantial growth in consumption of energy from renewable sources by its member countries and set a pathway to commitments under the 2015 Paris Agreement^2–4^. In 2018, renewable energy accounted for 18% of total final energy consumed in the EU-28, with its generation increasing by 64% over the previous 10 years. Renewable energy sources have central roles in the EU-28 electricity and heating-and-cooling sectors with shares in final consumption of 32% and 20%, respectively. Onshore wind (28%) was the leading source of renewable electricity, and solid biomass (83%) the dominant source of renewable heating-and-cooling in 2017^5^.

**Figure 1:**
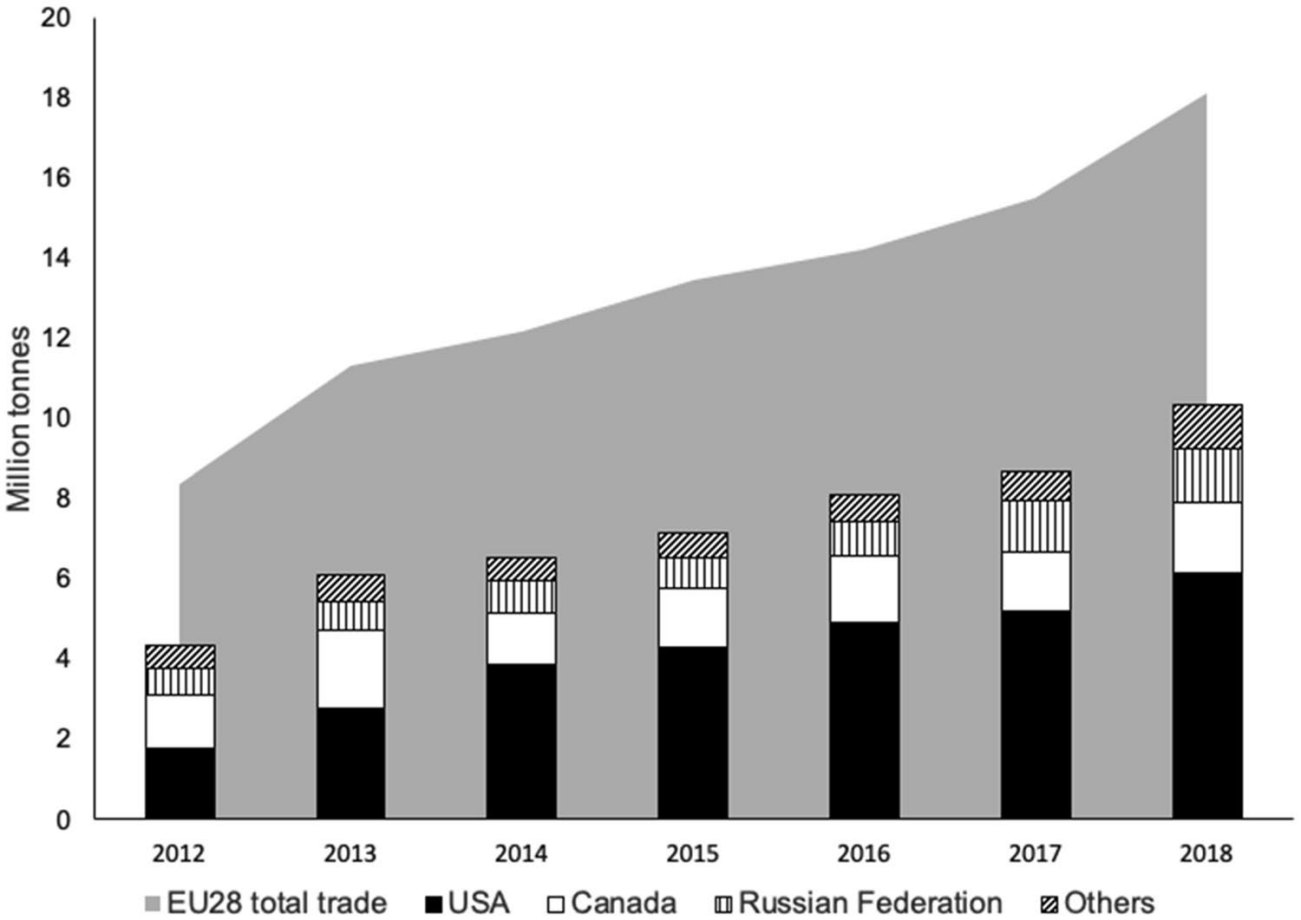
EU-28 wood pellet (commodity number: 440131) total trade and imports from top-three non-European partners^11^.

Under the EU RED, individual countries have developed National Action Plans to meet EU-wide goals tailored to particular resource availability and unique energy markets^6^. Former and current EU members such as the United Kingdom, Belgium, and Denmark rely heavily on imported biomass materials to meet their national renewable energy goals^7^. Wood fibers compressed into pellets emerged as an important type of commercially traded biofuel partly due to multiple factors. These included: fiber malleability, high energy content, relative ease of combustion, and existing supply-chains that facilitate competitive transportation and procurement costs further enabled by pellet standardization and environmental certification of forests^8–10^. The most recent statistics show that total trade of wood pellets across the EU-28 exceeded 18.2 million tons in 2018; 10.3 million tons were imported from non-European nations with the US as the largest trade partner^11,12^ (Fig. 1).

**Figure 2:**
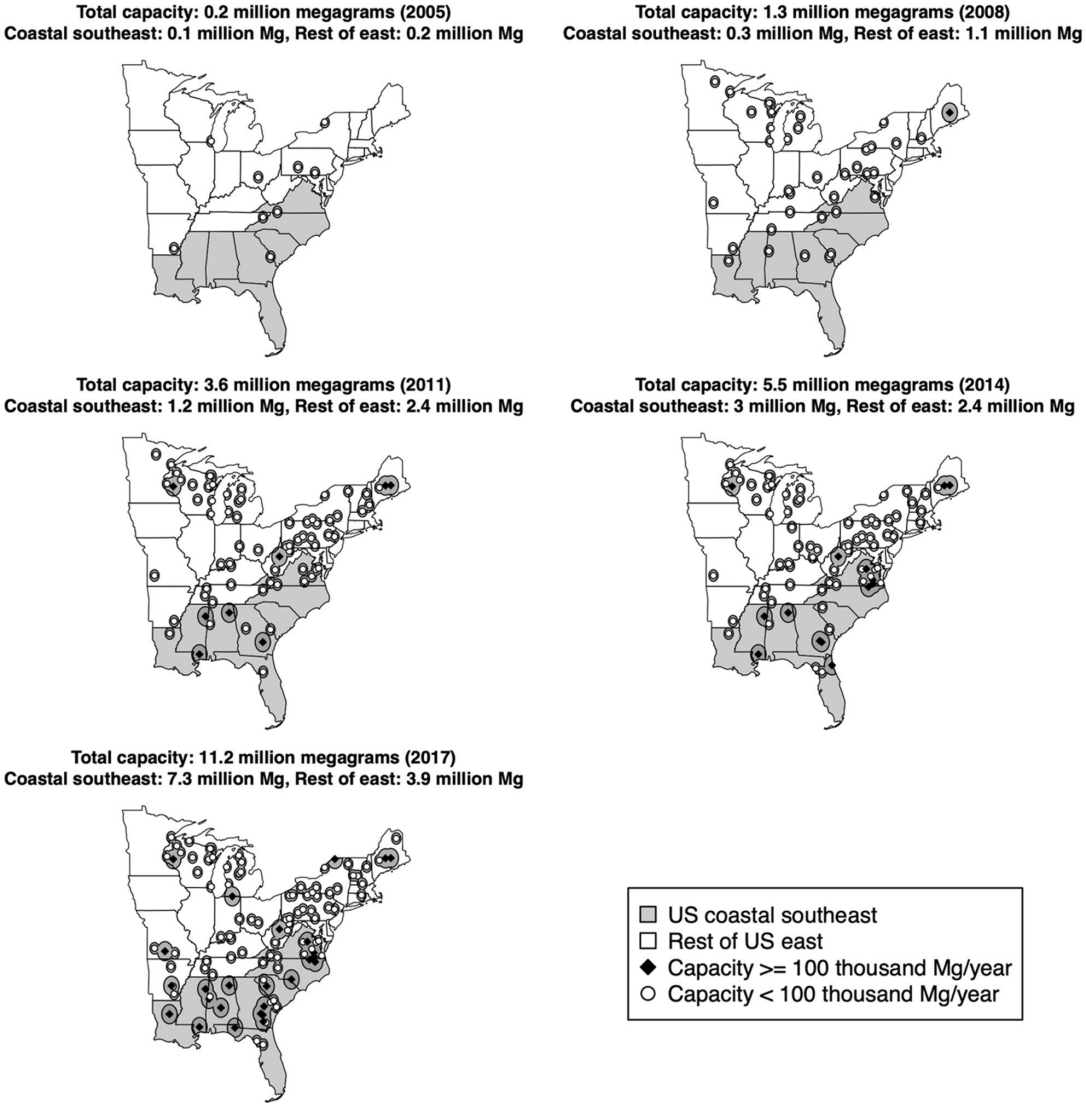
Location of wood pellet mills and corresponding procurement landscapes (concentric circles) in the eastern US distinguishing facilities of at least 100-thousand tons of annual installed capacity and region, 2005–2017. US coastal southeast includes the states: Alabama, Florida, Georgia, Louisiana, Mississippi, North Carolina, South Carolina, and Virginia. Maps generated in R using *sf* package^93,94^.

Implementation of EU RED National Action Plans triggered the establishment of new manufacturing facilities pelletizing wood fibers across the US coastal southeast^13,14^. It is important to note that EU RED National Action Plans do not target forests of any particular region, rather, the supply of US wood pellets developed organically partly due to cost competitive and comparative production advantages^15^. Prior to the adoption of EU RED in 2009, installed annual wood pellet manufacturing capacity along the coast of the southeastern US did not exceed 0.3 million tons but by 2017 it had expanded to 7.3 million tons (Fig. 2). Most recent estimates place total installed annual capacity in this region at 9.0 million tons^16^. Exponential growth in wood pellet manufacturing within the US coastal southeast has raised concerns over potential impacts on local forests. Insights into EU RED related policy effects on US forests have been inferred from a review of the extant literature^17,18^, case studies^14^, and land use projections^19,20^. Some argue that new demand for wood fibers is poised to cause major forestland losses or to degrade forests’ structure, composition and nutrient cycles^21,22^. Others posit that new energy markets could have a positive effect on forestland area by preventing deforestation and encouraging investments in multi-purpose tree plantations^13,23^.

The emergence of a resource-based manufacturing sector in direct response to market and public policy-driven demand offers an opportunity to assess associated changes in local natural resource conditions. During the same period following adoption of the EU RED, although of a lower magnitude, the number and capacity of wood pellet mills in the rest of the eastern US that exclusively supplies the domestic market also expanded^10,24^ (Fig. 2). Growth was partly the result of increased price competitiveness of wood pellets over heating oil and domestic policies promoting renewable heating^25^. The rise of the wood pellet industry in different areas responding to market and policy signals meets the general conditions for a natural experiment^26^. We assess changes in selected forest conditions for the US coastal southeast as compared to the rest of the eastern US to discern overall industry- associated effects and compare changes between them to infer EU RED-associated effects. Differences may be attributable to this policy intervention but, as in any other natural experiment, causality cannot be conclusive.

We follow the development of the wood pellet industry in the eastern US along with contemporaneous attributes of timberland (forestland capable of producing in excess of 1.4 m^3^ per ha per year not legally withdrawn from timber production, with a minimum area classification of 0.41 ha) structure and carbon (C). We georeference wood pellet mills and infer timberland conditions within mill-specific concentric radii that defined fiber procurement landscapes. We include (Table 1) three fundamental attributes for structure (number of live trees, number of growing-stock trees, number of standing-dead trees) and estimates of three carbon stocks (live trees, standing-dead trees, and soils). These attributes were estimated every 3 years between 2005 and 2017 using plot-level forest inventory data. Variation in timberland attributes within wood pellet mill procurement areas is modeled as a function of mill descriptors (initial operation, years of operation, manufacturing capacity) in addition to regional differences (coastal southeast and rest of eastern US), identification of period *post* US recession and of intensive domestic bioenergy policy interventions (cross-sections after year 2009), population (total inhabitants), access to export markets (within close proximity to ports exporting wood products), severe weather (extreme drought), and indicators of competition for wood fibers (overlap with procurement landscapes of wood-using power plants, pulp mills, other wood pellet mills). Econometric spatial panel estimation controls for interaction effects between regional, manufacturing size, and extreme weather covariates; idiosyncratic errors within each procurement area; and the potential for spatial autocorrelation. Detailed information on spatially-explicit covariates and estimation is presented in “Methods” section.

**Table 1:** Selected fundamental descriptors of timberland structure and carbon stocks within wood pellet industry procurement areas.

Descriptors	Description^a^
Structure: Number of live trees	Number of all live trees (at least 2.54 cm diameter at 1.37 m above the forest floor)
Structure: Number of growing-stock trees	Number of live large-diameter timber species (excludes non saw-log species) trees with one-third or more of the gross volume in the entire saw-log portion meeting grade, soundness, and size requirements or the potential to do so for medium-diameter and small-diameter trees. A growing-stock tree must have one 365.8 cm log or two noncontiguous 243.8 cm merchantable logs to qualify as growing stock
Structure: Number of standing-dead trees	Number of standing-dead trees (at least 12.7 cm in diameter at 1.37 m above the forest floor)
Carbon stocks: Live trees	Tons of carbon in aboveground and belowground (coarse root) biomass of ‘live trees’. The estimated volumes of wood and bark are converted to biomass based on the density of each. Additional components (e.g. tops, branches, and coarse roots, estimated according to adjusted component estimates)
Carbon stocks: Standing-dead trees	Tons of carbon in aboveground and belowground (coarse root) biomass of ‘standing dead trees’
Carbon stocks: Soils	Tons of carbon in organic soils estimated to a depth of 1 m

^a^Original data from the US Forest Service Forest Inventory Analysis Program^42^ reported in British Imperial units. Area-based estimates for timberlands within radii defining procurement areas derived after Bechtold et al.^59^. Timberland defined as forestland capable of producing in excess of 1.4 m^3^ per ha per year not legally withdrawn from timber production, with a minimum area classification of 0.41 ha.

Within the larger scope of industry-related impacts on forest resources, our *ex post* analysis of timberland structure and carbon stocks makes direct contributions to the extant literature by: (1) developing and implementing an analytical framework designed to assess changes in fundamental forest attributes of ecological, environmental, and commercial importance within industrial procurement landscapes; (2) identifying spatially-explicit renewable energy policy-induced effects on timberlands; (3) expanding the body of research assessing impacts of public policy interventions on forest conservation outcomes to actively managed forestlands; and (4) offering the first comprehensive and systematic assessment of EU RED-induced changes on US timberlands. Global expansion in national monitoring capacities^27^ offer the opportunity to comprehensively examine changes in fundamental forest attributes using a spatially-explicit analytical framework as we describe in this study. Our findings have direct implications for sustainable forest management, natural resource-based industries, and to the ongoing debate regarding the development of criteria and monitoring systems to deem biomass a sustainable and carbon neutral energy source^28^. Next, we describe our methods for the spatially-explicit analysis of timberland conditions within wood pellet mill procurement landscapes and industry-associated changes. We present and discuss our results first focusing on contemporaneous marginal changes in timberland conditions associated with wood pellet mill descriptors for annual operation, regional differences, and large manufacturing capacity. The latter helps discern significant associations linked to economies of scale needed to reach export markets^13,14^. We then discuss other influential factors related to anthropogenic, forest ecological region, and extreme weather effects.

## Methods

### Wood pellet industry and EU-induced effects on timberlands

**Figure 3:**
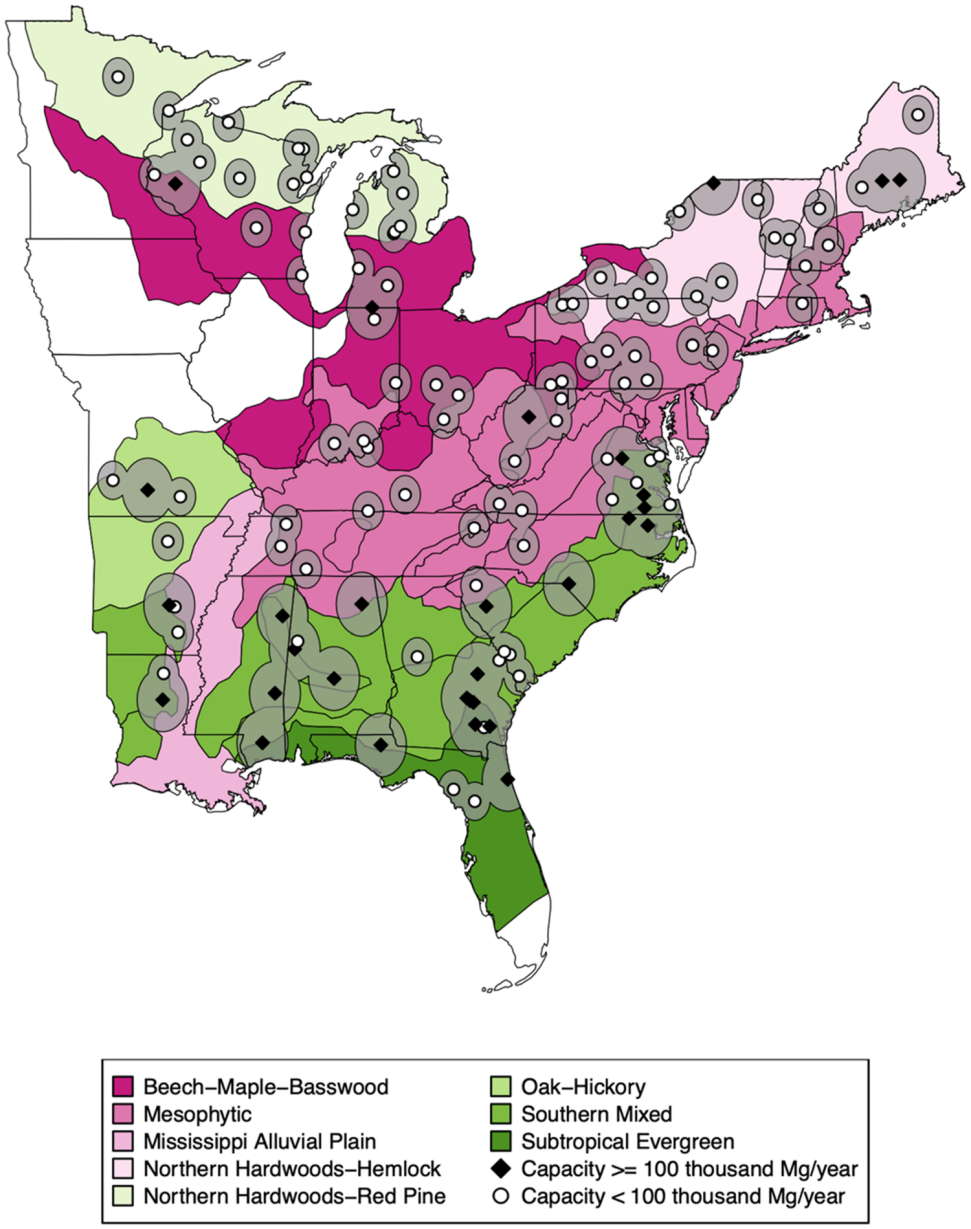
Forest ecological regions and centroids with corresponding procurement landscapes (concentric circles) of locations in the eastern US in which a wood pellet mill was operational between 2005 and 2017^35,81^. Maps generated in R using *sf* package^93,94^.

The establishment and operation of wood pellet mills can impact timberland conditions as wood fibers are sourced directly from forests or indirectly as a by-product of other wood-using industries^29^. New fiber demand can support the commercial flow of roundwood, pulpwood and logging residues procured directly from timberlands, while utilization of other wood product manufacturing residues can cause indirect land effects when creating markets for previously merchantable and non-merchantable materials^3^. The ecological footprint^30^ associated with procurement of low-cost raw materials is geographically delimited by transportation costs and a facility’s manufacturing capacity in line with established industry location theory principles^14,25,31^. For instance, in the short-term, mills of larger manufacturing capacity source raw materials from a larger procurement area. After a review of the current literature, a procurement radius of 80 km (50 British imperial miles) was assigned to facilities with a nominal annual manufacturing capacity of at least 100 thousand tons and a procurement radius of 48 km (30 British imperial miles) to those of lower capacity based on prevalent transportation distances and regional road tortuosity^32,33^. Moreover, wood fibers used for commercial purposes cannot originate from forestland legally withdrawn from timber production. These two criteria, proximity and timberland status, were used to define procurement landscapes for each of the 123 locations in the eastern US that at any point during our study period had an operational wood pellet mill. A procurement landscape parallels the definition of ‘sourcing area’ in RED II’s Article 2 as a “geographically defined area from which the forest biomass feedstock is sourced, from which reliable and independent information is available and where conditions are sufficiently homogeneous…”^34^. Information on wood pellet mill location, operational status, and manufacturing capacity was obtained from the most current and comprehensive database of US wood pellet mills available at the time^35^. As of March 2017, there were 123 wood pellet manufacturers in the eastern US that were operational at least 1 year between 2005 and 2017 (Fig. 3).

Evidence gathered from regional manufacturing and trade information shows that the totality of wood pellets exported to the EU are confined to facilities and ports within the US southeast coastal region^16,36^. Regional delimitation of EU RED-induced effects lent itself to the identification of a non-treated policy scenario to discern related impacts. We identified other states in the eastern US, which experienced a major expansion in wood pellet manufacturing over 2005–2017 period although in a lesser magnitude^18,37^, as a contrasting non-treated policy scenario (Fig. 2). Recent assessments of policy interventions on forest conditions have often relied on the statistical-based matching of policy treated and non-treated areas^38,39^ but such a statistically-based approach to identify counterfactual observations was not necessary in our case due to clarity between US regions responding to EU RED.

### Inferring timberland attributes within wood pellet mills’ procurement landscapes

We derived timberland conditions from plot-level information collected by the US Forest Service’s National Forest Inventory and Analysis (FIA) Program. Each plot covers an area of 674.48 m^2^ and is comprised of four equally-sized subplots each of 7.32 m radius. A plot is randomly located within each of the 24.28 km^2^ hexagons used to seamlessly sample the conterminous 48 US states. Full details on sampling design, data collection procedures, and attribute estimation are available in the FIA program documentation^40,41^. The FIA program applies remote and ground sampling to represent plot, condition, and tree measurements.

Timberland attributes were estimated for the 123 locations in the eastern US that at any point during our study period had an operational wood pellet mill. Timberland conditions assessed through selected attributes were estimated over 3-year windows between 2005 and 2017 from a total of 38,626 plots using the most recent available data from the FIA program^42^. Timberland attributes included number of live trees, number of growing-stock trees, number of standing-dead trees, above and belowground carbon in live trees (tons), above and belowground carbon in standing-dead trees (tons), and carbon in organic soil (tons). Chosen attributes reflected our interest in examining landscape level changes in structure (e.g. number of live trees), and addressing concerns over impacts on wildlife habitat (e.g. number of standing-dead trees) and major carbon pools (e.g., carbon above and belowground in standing live and dead trees, and soils). Area-based attributes were derived using data and SQL queries for area expansion factors from the FIA program^40,42,43^. Estimated average sampling errors for selected attributes were all less than 5%. Timberland attributes were accordingly estimated for the years 2005, 2008, 2011, 2014 and 2017 to construct our panel dataset. Supplementary Figure S1 shows box-plots for timberland attributes across all 123 observations by year and all 31 states in our study area. Supplementary Figure S2 shows average values of selected timberland attributes within procurement areas of wood pellet mills, and state-wide estimates, distinguishing between areas of the US coastal southeast and the rest of the eastern US. The study area encompasses a variety of forest conditions that allowed consideration of industrial biophysical factors influencing wood pellet mill procurement landscapes.

### Ex post assessment of timberland attributes associated with EU RED and other explanatory factors

Variation in selected timberland attributes was modeled as a function of variables triggered by implementation of EU RED and other factors that can explain changes in conditions within procurement areas of wood pellet mills. Explanatory factors included wood pellet mill descriptors, geographic region, competing industries, forest ecological regions, *post* recession and intensive domestic bioenergy policy period, population, and extreme weather events. Descriptive statistics for all covariates are disclosed in Table 2. Wood pellet mill descriptors included time-variant information on whether a new pellet mill was established and operational in a given period, the number of years it had been operational and whether the procurement area centroid was within 121 km (75 miles) of a waterway port exporting wood pellets^44^. Time-invariant mill descriptors included a category denoting the procurement area for a wood pellet mill of large manufacturing capacity (at least 100 thousand tons per year) to capture a larger footprint over the landscape reflected by correspondingly longer fiber sourcing radii; forest ecological region of largest expansion within a mill’s procurement landscape; and whether the procurement area centroid was within 121 km of a waterway port trading forest products. The latter helped control for any effects associated with wood procurement by industries other than the ones included in our covariates that can also trade via waterways^44^.

**Table 2:** Descriptions and descriptive statistics for model explanatory variables (*n* = 615).

Variable	Description	Min	Median	Mean	Max	SD
Mill present and operational	Dichotomous variable: 1 if a wood pellet mill was present and operating at corresponding year, 0 otherwise^35^. Its coefficient captures overall shifts associated with the operation of a wood pellet mill within procurement landscapes at any point during the 3-year sampling window	0.0	1.0	0.5	1.0	N/A
Years of operation	Number of years that a pellet mill has been in continuous operation^35^. Its coefficient captures changes associated with an additional year of wood pellet mill operation	0.0	1.0	3.2	28.0	4.4
Large manufacturing capacity	Dichotomous variable: 1 if wood pellet nominal annual capacity was at least 100 thousand tons, 0 otherwise^35^. Its coefficient controls for the larger procurement area associated with large-capacity pellet mills	0.0	0.0	0.2	1.0	N/A
Export market access	Dichotomous variable: 1 if the pellet plant was located within 121 km (75 British imperial miles) of a major port exporting forest products, 0 otherwise^35,37^. Its coefficient captures effects associated with access to export markets as inferred from the export of any forest product from a given port	0.0	1.0	0.5	1.0	N/A
US coastal southeast	Dichotomous variable: 1 if the pellet plant is in any of the US coastal southeastern states, 0 otherwise^35^. These include: Alabama, Florida, Georgia, Louisiana, Mississippi, North Carolina, South Carolina, and Virginia. Its coefficient captures any shifts in conditions within procurement areas of pellet mills in the US coastal southeast region as compared to the remainder of the eastern US	0.0	0.0	0.3	1.0	N/A
Forest ecological regions	Categorical variables: 1 corresponding to ecoregion of largest share within procurement landscape, 0 otherwise. Regions: Beech-Maple-Basswood, Mesophytic, Mississippi Alluvial Plain, Northern Hardwoods-Hemlock, Northern Hardwoods-Red Pine, Oak-Hickory, Southern Mixed, Subtropical Evergreen^81^. Coefficients capture level of changes in the forest region *versus* baseline Northern Hardwoods-Red Pine region	N/A	N/A	N/A	N/A	N/A
Wood pellet mills intersection	Continuous variable: Wood pellet procurement area intersected by corresponding procurement area of other wood pellet mills at each year (percent, greater than 1 if entire area overlap with more than once)^35^. Its coefficient captures any association between changes of greater levels of overlap in procurement areas	0.0	0.0	0.3	2.9	0.5
Wood-using power plants intersection	Area intersection between wood-using power plant procurement areas and base level at each year (percent)^35,82,83^ Its coefficient captures changes in pellet mills’ procurement landscapes associated with greater levels of intersection with the procurement for wood fibers to be used for electricity generation	0.0	1.1	1.4	6.2	1.2
Pulp mills intersection	Area intersection between pulp mill procurement areas and base level at each year (percent)^35,84–90^. Its coefficient captures changes in pellet mills’ procurement landscapes associated with greater levels of intersection with the procurement for wood fibers to be used by the pulp industry	0.0	0.6	1.1	10.8	1.5
US *post*-recession and intensive bioenergy policy period	Dichotomous variable: 1 for years 2011, 2014 and 2017, 0 otherwise. Its coefficient captures overall shifts in conditions associated with a *post*-recession period and any effects of domestic bioenergy policies	0.0	1.0	0.6	1.0	N/A
Population	Continuous variable: Population within a wood pellet plant procurement area (000 s)^91,92^. Its coefficient captures effects associated with changes in population within wood pellet mill procurement landscapes	21.4	298.3	486.5	2205.9	493.9
Extreme drought	Dichotomous variable: 1 if severe, extreme or exceptional drought reported for in at least 10% of the procurement area in August preceding a given year, 0 otherwisey^48,49^. Its coefficient captures any effects associated with the recent occurrence of extreme drought	0.0	0.0	0.1	1.0	N/A

EU RED contemporaneous associations with selected timberland attributes were determined by statistically significant differences between wood pellet mills’ procurement areas in the coastal southeast over the rest of the eastern US, and between larger- and smaller-scale facilities. The rest of the US eastern region outside the coastal southeast lent itself for statistical *ex post* comparison due to the presence of mixed conifer and broadleaf dominated landscapes, similar wood pelletization processes, comparable growth rates in industrial installed capacity, being located within the US facing the same federal laws and regulations, and consistency in forest inventory data collection^18,37^. However, differences exist as it is impossible to find perfect alternative scenarios to discern public policy impacts on natural resources^45^. Differences include non-identical forest ecosystems, different levels of industrial growth in recent years and a degree of heterogeneity in state-level forest policies^18^. The EU RED has reportedly influenced the establishment of larger wood pellet mills to benefit from economies of scale and to meet demand volumes, with exporting mills located near commercial ports for access to export routes^14^. Accordingly, differences between these descriptors and smaller manufacturing capacity helped discern for net EU RED policy effects.

Procurement landscapes for industries competing for similar wood fibers were defined and their contemporaneous degree of overlap with those of wood pellet mills were estimated. Competing industries included power plants consuming wood to generate electricity, and pulp mills. Radii for wood-using power plants and pulp mills were set at 80 km and 121 km to define commercial procurement areas for biopower and pulp industries, respectively^25,46^. We identified a period following the 2006–2009 recession of the US wood products industry to capture larger macroeconomic trends potentially omitted in other explanatory variables^47^. This *post* 2009 period coincides with a time of intensive US bioenergy public policy interventions, hence, this variable is also capturing any role associated with the expansion of related federal- and state-level programs^25^. Other explanatory factors were human population as a well-known element associated with changes in forest conditions, and a 1-year lagged variable identifying procurement areas affected by extreme drought^48,49^. See Supplementary Notes 1 and 2, and Figure S3 for details on the estimation of values for population and procurement area intersection to gauge degree of industry competition.

The issue of confounding can challenge empirical assessments of public policy impacts. A potentially prominent confounding effect mentioned in the recent literature on policy interventions and forest conditions is that of anticipatory behavior^38^ which could involve tree harvesting in expectation of policy implementation^50^. However, anticipatory behavior in the form of preemptive timber harvesting is unlikely to be an issue in this assessment. Any type of harvesting associated with the supply of wood fibers for pellets only occurs concurrent with the beginning of manufacturing operations. While anticipatory timber harvesting would be a rare occurrence in the context of our study area, wood pellet mills can be deliberately located within areas that are already endowed with suitable standing timber (which explains the common use of the term ‘woodsheds’^14^). Hence, we attempted to control for initial conditions within wood pellet mill procurement landscapes in our econometric analysis by identifying whether in any of our time periods a pellet mill was operational. We then examine annualized contemporaneous changes by including the length of milling operations. Finally, the localized nature of wood fiber markets motivated the adoption of an econometric model that controls for spatially-explicit and idiosyncratic conditions within procurement areas.

### Spatial econometric panel analysis

A model was specified to *ex post* examine changes in the $Y$ th timberland attribute across $N$ observations over $t$ periods for landscapes where at any point between 2005 and 2017 a wood pellet mill was operational (Eq. 1). ${Y}_{N,t}$, the $N\times 1$ vector of observations of the dependent variable in period year $t$, was modeled as a function of an overall parameter intercept (*α*); $N\times 2$ matrix of wood pellet mill time-variant variables ${X}_{N,t}$ inclusive of mills being in operation in year *t*, number of years of operation at year *t*; $N\times 10$ matrix ${F}_{N}$ of time-invariant descriptors denoting access to forest products ports, mill annual manufacturing capacity (at least 100 thousand tons), coastal southeast areas, and forest ecological regions; $N\times 3$ matrix ${C}_{N,t}$ of time-varying degrees of overlap in procurement areas of wood pellets mills, electricity-generating plants using wood as an energy feedstock, and pulp mills; vectors of 2011–2017 *post* recession period ${R}_{N,t}$, population ${P}_{N,t}$, 1-year lagged variable for extreme drought ${D}_{N,t-1}$ for $N$ observations over period year $t$; and finally $N\times 11$ matrix of interaction terms ${INT}_{N,t}$ between wood pellet mills presence, coastal southeastern location and years of operation, large manufacturing capacity, and extreme weather, across observations and years. Extreme drought was included as a 1-year lagged variable to correctly infer associations due to recording of drought in the summer months and surveying of forest plots throughout the year. Moreover, this timeframe allowed for the expression of any response between extreme drought and changes in timberland attributes to occur. We also included the $N\times 1$ vector of location-specific disturbance term ${u}_{N,t}$ including ${W}_{N}$, an $N\times N$ time-invariant weight matrix of row-normalized inverse distances between procurement landscape centroids, $\rho$ a scalar autoregressive parameter, and ${\varepsilon }_{N,t}$ a $N\times 1$ vector of period $t$ random effects^25,51–53^. It can be shown that the disturbance vector ${u}_{N,t}$ can be uniquely solved in terms of the random effects vector ${\varepsilon }_{N,t}$^53,54^. As such, a spatial model was estimated as:

1 $${Y}_{N, t}=\alpha +{X}_{N,t}{\beta }_{1}+{F}_{N}{\beta }_{2}+{C}_{N,t}{\beta }_{3}+{R}_{N, t>2009}{\beta }_{4}+{P}_{N,t}{\beta }_{5}+{D}_{N,t-1}{\beta }_{6}+ {INT}_{N, t}{\beta }_{7}+{u}_{N,t},$$

and

2 $${u}_{N,t}={\left({I}_{N}-\rho {W}_{N}\right)}^{-1}{\varepsilon }_{N,t} ,$$

where $N=123$, $t periods=\{2005, 2008, 2011, 2014, 2017\}$, and *β*s correspond to $K\times 1$ vectors of the respective explanatory variables. ${W}_{N}$ (Eq. 2) is a row-normalized weight matrix of the inverse distances between pellet mills ${\omega }_{N}$ truncated at a distance of 161 km (100 British imperial miles)^8^ as the limit for spatial autocorrelation such that:

3 $${\omega }_{N}=\left\{\begin{array}{l}\frac{1}{{d}_{ij}},\quad {d}_{ij}<161km\\ 0,\quad otherwise\end{array}\right., i,j=\mathrm{1,2},\dots ,123 \; and\;  i\ne j$$

The random effects error vector ${\varepsilon }_{N,t} \sim IID(0,{\sigma }_{\varepsilon }^{2})$ including unobserved idiosyncratic procurement area effects, and cross-sectional fixed and time-variant components, were tested for correlation with explanatory variables for correct estimation as a fixed or random model. Hausman test^55^ results (see Supplementary Note 3 and Table S1) favored a random effects specification, hence, the spatial panel regression with random effects was estimated using maximum likelihood^52^. *β* coefficients were standardized to estimate relative association of the *i*th covariate on dependent variable *y* to ease comparison across models as follows^51^:

4 $$Standardized \; {\beta }_{i}= {\beta }_{i} \frac{{S}_{{x}_{i}}}{{S}_{y}}$$

where the S_x_ and S_y_ correspond to the sample standard deviations for the covariate and dependent variable, respectively. Coefficients were also adjusted to calculate average hectare-level effects within procurement areas to afford direct interpretation and comparability across models (see Supplementary Note 4 for details on conversion followed to derive associations from coefficients to area-adjusted effects).

### Interpretation of coefficients and wood pellet industry-related associations

The linear nature of our panel regression allows for the direct interpretation of explanatory variables’ parameterized associations with particular timberland attributes, else constant. Here we offer a few examples to clarify their interpretation in relation to inference of wood pellet industry associations.

- Overall timberland conditions: The intercept parameter α controls for overall baseline timberland conditions across all wood pellet mill procurement landscapes irrespective of regional location or if pelletization operations have started. The parameter for ‘*Large manufacturing capacity*’ captures an overall shift in conditions associated with larger procurement areas. As expected, α intercept and larger procurement area *β* parameters are strongly statistically significant from zero [*p* value < 0.001]. The parameter for the ‘*Coastal southeast region’* captures overall differences between procurement areas in the coastal southeast and the rest of the eastern US. Coastal southeast regional differences specifically associated with wood pellet mill presence and operation, years of operation, large manufacturing capacity, export market access, extreme drought are captured in corresponding interaction terms.
- Initial wood pellet industry associated timberland conditions: Any differences in conditions at the initiation of wood pellet mill operations is captured in the parameter for ‘*Mill present and operational*’. Initial differences in timberland conditions within procurement areas of the coastal southeast, as compared with those in the rest of the US east, are captured in the parameter for variable ‘*Pellet mill present and operational* × *coastal southeast region*’.
- Wood pellet industry-related temporal changes in timberland conditions: The parameters and respective interactions for the variable ‘*Years of operation*’ capture differences in timberland conditions associated with an additional year of operation. Any significant deviations from this trend within procurement landscapes of wood pellet mills in the coastal southeast and of large capacity mills are captured in the coefficients for ‘*Years of operation* × *coastal southeast region’* and ‘*Years of operation* × *large manufacturing capacity’*, respectively.

## Results

**Figure 4:**
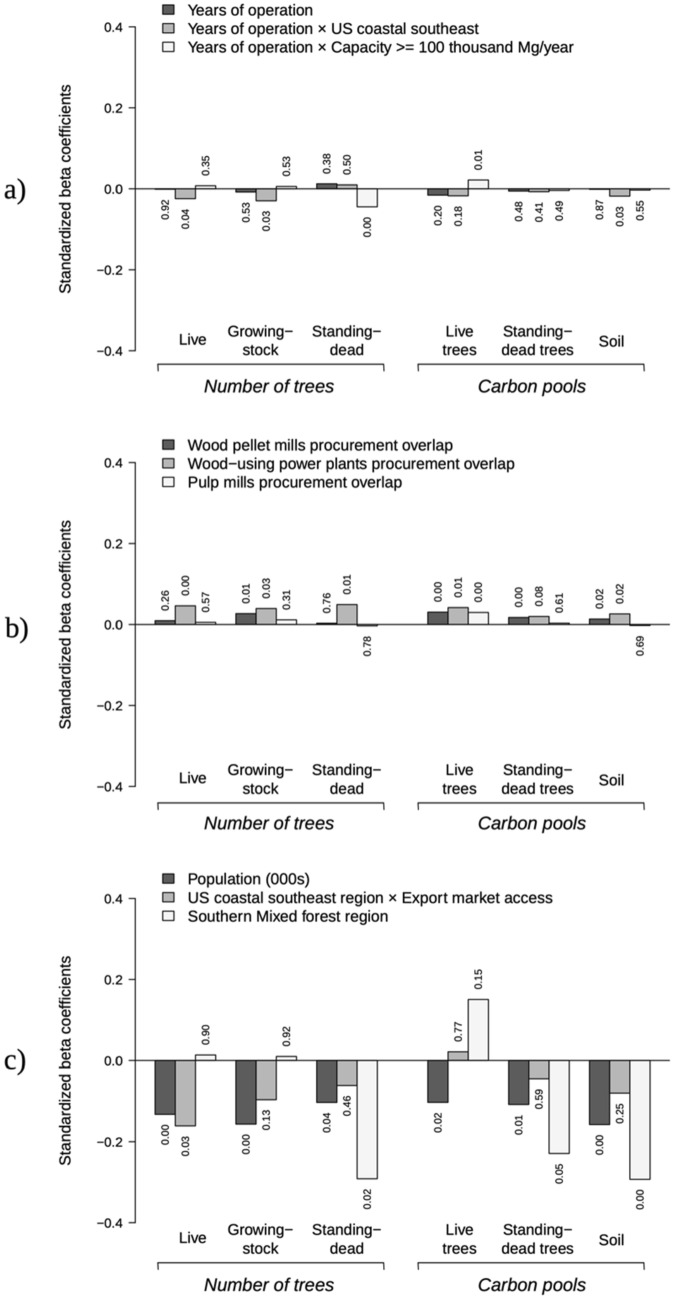
Standardized coefficients and *p value*s for (**a**) variables capturing annual effects of wood pellet mill operation interacted with US coastal southeast region and large-scale manufacturing categories, (**b**) fiber competition from power, pulp operations and other wood product factors, and (**c**) population, forest product exporting access from US coastal southeast, and southern mixed forest ecological region. Standardized coefficients represent the expected change (standard deviation) of selected timberland descriptors and carbon stocks associated with one standard deviation change in a given covariate.

Estimated regression coefficients, area-adjusted marginal effects, and *p* values from the random-effects spatial panel model are presented in Tables 3 and 4. Standardized coefficients, to denote the relative importance of selected covariates on changes in fundamental timberland attributes, are included in Fig. 4. Coefficients that tested the association between wood pellet manufacturing and descriptors linked to EU RED, US coastal southeast and large-scale manufacturing, exhibited several statistically-significant associations (Fig. 4a). Compared with procurement areas of wood pellet mills in the rest of the eastern US, the operation of wood pellet plants in the US coastal southeast over time was associated with fewer numbers of live (*p* = 0.04) and growing-stock trees (*p* = 0.02) and lower carbon pools in soils (*p* = 0.03) to the rate of 8.21 ± 3.97 million trees and 1.55 ± 0.69 million trees, and 234.95 ± 105.97 thousand tons of carbon per procurement area per year (Table 3). These are the equivalent of 11.22 and 2.12 trees/ha/year, and 0.32 tons C/ha/year, respectively. Among large-scale facilities, their annual operation was associated with a decreasing trend in the number of standing-dead trees (*p* = 0.00) and more C stocks in live trees (*p* = 0.01). Average associations were found at the rate of 0.23 fewer standing-dead trees/ha/year, and 0.20 more tons C/ha/year in live-tree pools.

**Table 3:** Panel regression coefficients of wood pellet mill descriptors and interaction terms, along area-adjusted marginal effects (*n* = 615).

	Regression coefficients and area-adjusted effects on timberland attributes											
	Number of live trees (trees)		Number of growing-stock trees (trees)		Number of standing-dead trees (trees)		Carbon in live trees (tons)		Carbon in standing-dead trees (tons)		Carbon in soil (tons)	
	Coefficient [std error]	Ha-adj [*p value*]	Coefficient [std error]	Ha-adj [*p value*]	Coefficient [*p value*]	Ha-adj [*p value*]	Coefficient [std error]	Ha-adj [*p value*]	Coefficient [std error]	Ha-adj [*p value*]	Coefficient [std error]	Ha-adj [*p value*]
**Wood pellet mill descriptors**												
Mill present and operational	− 13,708,018 [16212211]	− 18.719 [0.398]	− 2,067,379 [2851538]	− 2.823 [0.468]	12,707 [300346]	0.017 [0.966]	− 43,798 [473636]	− 0.060 [0.926]	4292 [17715]	0.006 [0.809]	7396 [430405]	0.010 [0.986]
Years of operation	− 231,315 [2371992]	− 0.316 [0.922]	− 260,815 [419740]	− 0.356 [0.534]	39,023 [44467]	0.053 [0.380]	− 87,141 [68017]	− 0.119 [0.200]	− 1781 [2546]	− 0.002 [0.484]	− 10,169 [62931]	− 0.014 [0.872]
Large manufacturing capacity (> 100 thousand tons/year)	1,167,192,244 [153055021]	896.551 [< 0.001]	202,804,736 [19873853]	155.780 [< 0.001]	25,148,827 [2508366]	19.317 [< 0.001]	41,293,846 [4116842]	31.719 [< 0.001]	2,938,088 [265809]	2.257 [< 0.001]	46,217,599 [5617629]	35.501 [< 0.001]
Export market access	56,985,291 [89552582]	77.817 [0.525]	− 430,840 [11751836]	− 0.588 [0.971]	258,982 [1481477]	0.354 [0.861]	− 2,553,604 [2386620]	− 3.487 [0.285]	11,976 [153521]	0.016 [0.938]	112,568 [3278341]	0.154 [0.973]
US coastal southeast region	225,149,136 [180034738]	307.454 [0.211]	1,430,151 [24313504]	1.953 [0.953]	− 1,020,655 [3057966]	− 1.394 [0.739]	− 4,146,359 [4678017]	− 5.662 [0.375]	− 38,769 [297825]	− 0.053 [0.896]	2,568,086 [6529394]	3.507 [0.694]
**Coastal southeast, size and weather interactions**												
Years of operation × US coastal southeast region	− 8,214,812 [3968698]	− 11.218 [0.038]	− 1,550,914 [692630]	− 2.118 [0.025]	49,514 [73554]	0.068 [0.501]	− 155,133 [115088]	− 0.212 [0.178]	− 3581 [4349]	− 0.005 [0.410]	− 234,946 [105967]	− 0.321 [0.027]
Years of operation × large manufacturing capacity	5,288,731 [5693026]	2.600 [0.353]	623,006 [1001992]	0.306 [0.534]	− 474,920 [105573]	− 0.233 [< 0.001]	407,846 [166049]	0.200 [0.014]	− 4303 [6209]	− 0.002 [0.488]	− 89,386 [151109]	− 0.044 [0.554]
Pellet mill present and operational × large manufacturing capacity	30,525,058 [28196286]	15.006 [0.279]	12,354,666 [4928202]	6.074 [0.012]	1,265,000 [518979]	0.622 [0.015]	3,980,587 [829492]	1.957 [< 0.001]	101,917 [31078]	0.050 [0.001]	1,654,599 [749911]	0.813 [0.027]
Pellet mill present and operational × US coastal southeast region	− 11,343,583 [23520244]	− 15.490 [0.630]	− 817,645 [4134392]	− 1.117 [0.843]	− 473,292 [435130]	− 0.646 [0.277]	− 151,285 [688530]	− 0.207 [0.826]	− 26,229 [25755]	− 0.036 [0.308]	− 353,588 [624461]	− 0.483 [0.571]
Pellet mill present and operational × export market access	29,415,161 [16816968]	40.168 [0.080]	3,693,459 [2958086]	5.044 [0.212]	198,237 [311440]	0.271 [0.524]	− 522,333 [491585]	− 0.713 [0.288]	6301 [18367]	0.009 [0.732]	264,525 [446207]	0.361 [0.553]
Large manufacturing capacity × US coastal southeast region	272,872,617 [176224109]	134.144 [0.122]	70,630,728 [23102816]	34.722 [0.002]	− 12,902,857 [2917220]	− 6.343 [< 0.001]	9,948,973 [4685736]	4.891 [0.034]	− 1,107,071 [302149]	− 0.544 [< 0.001]	11,903,191 [6457605]	5.852 [0.065]
Large manufacturing capacity × export market access	303,984,583 [181285546]	149.439 [0.094]	17,071,330 [23877822]	8.392 [0.475]	3,269,215 [3015937]	1.607 [0.278]	− 6,538,822 [4794995]	− 3.214 [0.173]	72,370 [308505]	0.036 [0.815]	8,467,155 [6629903]	4.162 [0.202]
Large manufacturing capacity × Extreme drought	18,552,930 [28838903]	9.121 [0.520]	2,494,171 [5021558]	1.226 [0.619]	− 743,535 [528612]	− 0.366 [0.160]	591,850 [852772]	0.291 [0.488]	− 22,271 [31982]	− 0.011 [0.486]	− 170,210 [767704]	− 0.084 [0.825]
US coastal southeast region × export market access	− 405,494,006 [187092426]	− 553.725 [0.030]	− 37,856,269 [25159577]	− 51.695 [0.132]	− 2,340,308 [3171420]	− 3.196 [0.461]	1,436,162 [4862017]	1.961 [0.768]	− 167,417 [310042]	− 0.229 [0.589]	− 7,775,362 [6791209]	− 10.618 [0.252]
US coastal southeast region × extreme drought	73,931,236 [35193338]	100.957 [0.036]	9,152,187 [6338873]	12.498 [0.149]	1,931,192 [666057]	2.637 [0.004]	1,033,485 [1007470]	1.411 [0.305]	80,751 [37256]	0.110 [0.030]	1,606,212 925923]	2.193 [0.083]
Export market access × extreme drought	5,507,711 [26777221]	7.521 [0.837]	103,830 [4734887]	0.142 [0.983]	973,032 [498292]	1.329 [0.051]	579,116 [778860]	0.791 [0.457]	36,014 [29019]	0.049 [0.215]	630,684 [709029]	0.861 [0.374]

We found discernible trends on timberland conditions within procurement landscapes that overlapped with other pellet mills as well as with other industries competing for wood fibers (Fig. 4b). A one percentage-point increase in overlap of wood pellet mill procurement areas, denoting greater competition, was associated with larger C stocks in above and belowground live (2.07 tons C/ha; *p* = 0.00) and standing-dead (0.06 tons C/ha; *p* = 0.00) trees pools, and in soils (1.32 tons C/ha; *p* = 0.02). A direct association was also found between greater overlap in procurement areas and a larger number of growing-stock trees. All selected timberland conditions showed statistically significant (*p* < 0.05) positive associations with the greater overlap of procurement areas between wood pellet mills and power plants using biomass for electricity generation. The overlap between wood pellet mill procurement landscapes with those of pulp mills was associated with an increase in carbon within live tree pools (0.68 tons/ha; *p* = 0.00) possibly as a result of land management that incentivizes growing wood fibers within procurement distances (Table 4).

**Table 4:** Regression coefficients of competition, domestic energy policy, population, extreme weather, and forest ecological regions, along area-adjusted marginal effects (*n* = 615).

	Regression coefficients and area-adjusted effects on timberland attributes											
	Number of live trees (trees)		Number of growing-stock trees (trees)		Number of standing-dead trees (trees)		Carbon in live trees (tons)		Carbon in standing-dead trees (tons)		Carbon in soil (tons)	
	Coefficient [std error]	Ha-adj [*p value*]	Coefficient [std error]	Ha-adj [*p value*]	Coefficient [*p value*]	Ha-adj [*p value*]	Coefficient [std error]	Ha-adj [*p value*]	Coefficient [std error]	Ha-adj [*p value*]	Coefficient [std error]	Ha-adj [*p value*]
Intercept	723,714,402 [160215807]	988.273 [< 0.001]	159,168,103 [23294108]	217.353 [< 0.001]	16,381,111 [2912528]	22.369 [< 0.001]	20,158,045 [3931331]	27.527 [< 0.001]	1,811,028 [243218]	2.473 [< 0.001]	60,736,726 [5667301]	82.939 [< 0.001]
**Competition for wood fibers**												
Pellet mills intersection	17,623,043 [15533514]	24.065 [0.257]	7,817,897 [2845724]	10.676 [0.006]	90,423 [299495]	0.123 [0.763]	1,518,168 [435888]	2.073 [< 0.001]	47,944 [16064]	0.065 [0.003]	965,974 [406900]	1.319 [0.018]
Wood-using power plants intersection	33,792,783 [11533030]	46.146 [0.003]	4,515,712 [2045643]	6.166 [0.027]	547,555 [219187]	0.748 [0.012]	817,378 [322872]	1.116 [0.011]	21,675 [12251]	0.030 [0.077]	739,658 [307636]	1.010 [0.016]
Pulp mills intersection	3,345,170 [5882480]	4.568 [0.570]	1,116,992 [1096873]	1.525 [0.309]	− 32,230 [115392]	− 0.044 [0.780]	494,876 [162514]	0.676 [0.002]	3069 [5962]	0.004 [0.607]	− 60,313 [153317]	− 0.082 [0.694]
**Domestic energy policy, population, extreme weather**												
US post-recession and intensive bioenergy policy period (year > 2009)	1,995,419 [16076204]	2.725 [0.901]	− 175,081 [3050677]	− 0.239 [0.954]	86,257 [320346]	0.118 [0.788]	2,659,294 [438870]	3.631 [< 0.001]	42,141 [16109]	0.058 [0.009]	647,168 [418066]	0.884 [0.122]
Population (000 s)	− 244,614 [82006]	− 0.334 [0.003]	− 45,087 [11423]	− 0.062 [< 0.001]	− 2885 [1407]	− 0.004 [0.040]	− 5066 [2179]	− 0.007 [0.020]	− 297 [122]	0.000 [0.015]	− 11,218 [2761]	− 0.015 [< 0.001]
Extreme drought	− 17,906,164 [27373777]	− 24.452 [0.513]	− 1,373,105 [4955851]	− 1.875 [0.782]	− 1,092,485 [520787]	− 1.492 [0.036]	− 212,834 [779295]	− 0.291 [0.785]	− 34,943 [28769]	− 0.048 [0.225]	− 420,881 [719058]	− 0.575 [0.558]
**Forest ecological regions**												
Beech-Maple-Basswood	− 434,710,686 [184579792]	− 593.622 [0.019]	− 75,142,092 [25580012]	− 102.611 [0.003]	− 8,042,965 [3219061]	− 10.983 [0.012]	− 4,118,210 [4672821]	− 5.624 [0.378]	− 917,191 [293509]	− 1.252 [0.002]	− 35,739,930 [6625278]	− 48.805 [< 0.001]
Mesophytic	− 135,855,963 [184419117]	− 185.519 [0.461]	− 15,624,705 [26227794]	− 21.336 [0.551]	− 3,126,833 [3296784]	− 4.270 [0.343]	15,421,205 [4563267]	21.059 [0.001]	− 75,140 [284419]	− 0.103 [0.792]	− 34,008,447 [6570662]	− 46.440 [< 0.001]
Mississippi alluvial plain	− 51,707,490 [405951333]	− 70.61 [0.899]	− 45,925,699 [54636799]	− 62.714 [0.401]	1,976,089 [6896097]	2.698 [0.774]	− 2,261,913 [10537815]	− 3.089 [0.830]	− 205,902 [675020]	− 0.281 [0.760]	− 20,477,039 [14767846]	− 27.963 [0.166]
Northern Hardwoods-Hemlock	387,433,282 [203421948]	529.062 [0.057]	22,674,120 [29341154]	30.963 [0.440]	9,494,009 [3687470]	12.965 [0.010]	12,570,594 [4965414]	17.166 [0.011]	1,007,971 [309839]	1.376 [0.001]	− 24,287,135 [7232669]	− 33.165 [0.001]
Oak-Hickory	− 191,030,406 [365169938]	− 260.863 [0.601]	− 52,173,345 [54308603]	− 71.246 [0.337]	− 7,088,654 [6808037]	− 9.680 [0.298]	− 2,188,873 [8730600]	− 2.989 [0.802]	− 720,672 [540062]	− 0.984 [0.182]	− 45,093,674 [12867697]	− 61.578 [< 0.001]
Southern Mixed	28,340,564 [231841790]	38.701 [0.903]	3,212,732 [32393038]	4.387 [0.921]	− 9,277,032 [4078627]	− 12.668 [0.023]	8,430,446 [5825176]	11.512 [0.148]	− 715,236 [367928]	− 0.977 [0.052]	− 23,687,273 [8330909]	− 32.346 [0.004]
Subtropical Evergreen	− 344,202,116 [325991994]	− 470.028 [0.291]	− 5,342,104 [46029757]	− 7.295 [0.908]	− 10,307,834 [5791323]	− 14.076 [0.075]	− 1,821,431 [8109876]	− 2.487 [0.822]	− 1,267,733 [509901]	− 1.731 [0.013]	− 8,485,421 [11671019]	− 11.587 [0.467]

Across all models, coefficients capturing marginal effects of population and close proximity to a forest product exporting port in the US coastal southeast had some of the largest absolute and standardized values (Fig. 4c). Population levels were inversely associated with all timberland descriptors used in our analysis. Procurement areas that had 1000 more inhabitants than the average had 244.61 ± 82.01 thousand fewer live trees, 45.09 ± 11.42 thousand fewer growing-stock trees, 2.88 ± 1.41 thousand fewer standing-dead trees, as well as 5.07 ± 2.18 thousand fewer tons of C in live trees, 297 ± 122 fewer tons of C in standing-dead trees and 11.22 ± 2.76 thousand fewer tons of C in soils in a given year. Wood pellet mill procurement landscapes within close proximity (i.e. 121 km) to a forest product exporting port in the US coastal southeast had 405.49 ± 187.09 million fewer live trees than those in the rest of the US east at a rate of − 553.72 trees/ha (*p* = 0.03). Comparatively, the standardized average marginal association of population with lower carbon pools in soils was 53-fold greater than that of annual operation of a large-scale wood pellet mill (Fig. 4a,b). This highlights the greater extent to which population can influence changes in certain timberland attributes than does the wood products industry. We also mention the importance of controlling for overall ecological conditions where wood pellet mils are located. For instance, the Oak-Hickory forest ecoregion showed statistically significant mean differences in nearly all timberland attributes (Fig. 4c).

**Figure 5:**
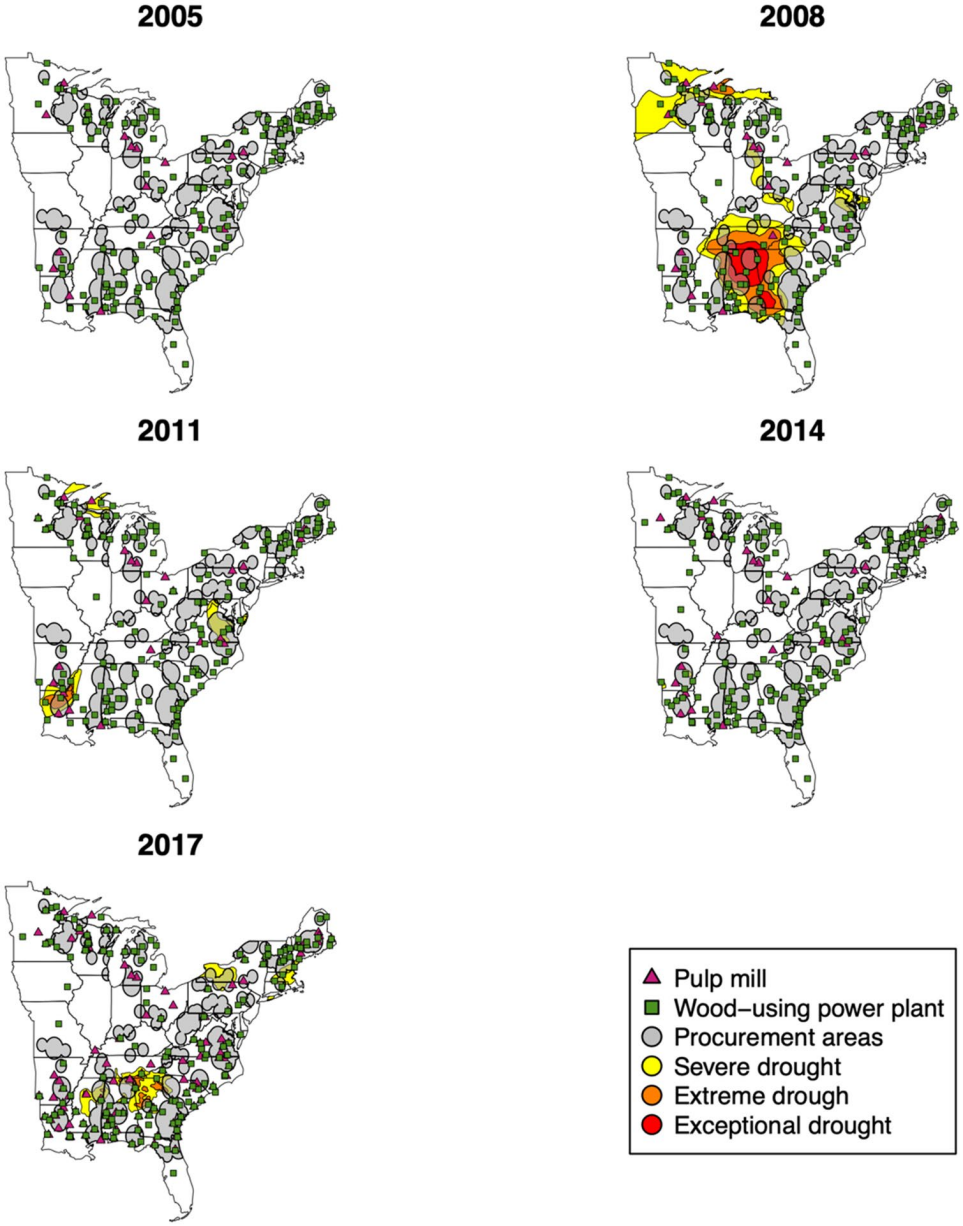
Location of wood-using power plants, pulp mills, and areas of reported extreme weather conditions (lagged 1 year), 2005–2017. Maps generated in R using *sf* package^93,94^.

Coefficients capturing extreme drought effects on selected timberland conditions showed some statistically significant associations. Extreme drought (Fig. 5) was associated with more live and standing-dead trees, as well as an increase in C stocks in standing dead-trees within procurement areas of the US coastal southeast. The ecological relationships leading to this observation may not be intuitive. A larger number of live trees might be explained by resprouting, and more standing-dead trees and C found in dead and soils pools as a consequence to higher mortality rates^56^. It is worth mentioning that differences in localized drought effects might also be influenced by recent precipitation events, among other contemporary and legacy factors, such as species composition and soil characteristics (e.g. parental material, depth).

## Discussion

### EU RED and US coastal southeast timberland conditions

The EU recognizes environmental risks associated with greater demand for biomass for energy and has issued non-binding recommendations on biomass sustainability criteria to meet EU RED targets^57^. The European Commission requires monitoring so that biomass used for energy, regardless of origin, does not cause deforestation or degradation of habitats^3,58^. In order to ensure compliance with EU-wide sustainability recommendations and any EU member-level sustainability criteria, the Sustainable Biomass Program (SBP) emerged as a private certification system designed to evaluate the legal and sustainable sourcing of wood pellets and chips used in large-scale energy generation^59^. It relies heavily on existing sustainable forest management and chain-of-custody certificate programs such as the Forest Stewardship Council and the Programme for Endorsement of Forest Certification. In addition to forest management and product chain-of-custody compliance, SBP certification offers an assessment of risk associated with feedstock in regards to legality and sustainability compliance and methods to collect and communicate data along the supply chain inclusive of greenhouse gas budgets.

Results identify diverse trends within procurement landscapes of pellet mills of the US coastal southeast from which wood pellets are exported to the EU. Fewer numbers of live and growing-stock trees with no decline in their corresponding C pools likely reflect on land management practices that favor growth in merchantable trees denoting more intensive silvicultural treatments. This trend can be explained by how new demand for wood pellets creates market incentives to grow and regrow wood fibers^13,19^. Moreover, this trend for greater demand of fibers sourced directly from timberland is reflected on the composition of wood fibers being pelletized. National industry surveys show fiber types used in wood pellet production were largely comprised of sawmill residues (69%, by weight)^10^ prior to EU RED implementation. Sawmill residues accounted for about 18% of fibers used in wood pellet manufacturing by 2017^16^. Most fibers (49%) came from residual biomass of little to no commercial value (e.g., bark, logging residues, wood chips, post-consumer wood, unmerchantable wood), and roundwood/pulpwood (20%). The remaining fibers (13%) were sourced from residues of wood product manufacturing.

A fewer number of standing-dead trees over time within procurement areas of large-scale wood pellet mills might also be an indication of more intensive management practices that remove previously non-commercial biomass. This trend had been previously reported within the procurement area for an exporting wood pellet mill in the state of Georgia^14^. This result is of direct relevance to policy makers as the expansion of international markets might make large-scale wood pellet manufacturing facilities more prevalent. Some have argued that management that removes dead biomass prone to ignition could reduce the risk and intensity of wildfires, hence, it might point to potentially desirable outcomes^60^. However, it is important to note that woody biomass extraction continuously exceeding natural disturbances has been linked with the disruption of nutrient cycles to the detriment of site productivity and saproxylic communities, and could also negatively impact ecosystem structure as it relates to wildlife habitat and forest biodiversity^61–63^. There were also negative discernible effects on carbon stocks in soil pools associated with the annual continued operation of wood pellet facilities in the coastal southeast (Table 3). Our results point to a flux in C stocks across different pools in procurement areas of large-scale wood pellet mills of the US coastal southeast. The former descriptor was associated with higher C levels in live trees (407.85 ± 166.05 thousand tons of C), while the latter exhibited less C in soil pools (234.95 ± 105.97 thousand tons of C) per procurement area. On the balance, there has been a net contemporaneous positive effect.

It is worth noting that our *ex post* analysis of systematic changes in timberland conditions is confined to the boundaries for current commercial procurement distances. Issues of leakage beyond procurement areas are not addressed in this research. For instance, it is plausible that growing wood pellet demand had spillover effects to lands well outside mills’ procurement radii by shifting competition for similar fibers to new supply areas. Further, our results are limited to the time period evaluated (2005–2017) with EU RED targets instituted in 2009 and sustainability recommendations first adopted in 2015. Within this timeline of public policy interventions and commercial timberland management rotations, and given the most recently available forest inventory data, our analysis of 12 years is still limited in its ability to identify longer-term shifts in timberland conditions and C pools. In addition to the core attributes presented in this manuscript to assess changes within procurement landscapes, we recommend to also study systematic changes in biodiversity indicators.

On 13 March 2019 the European Commission proposed to the European Parliament the formal assessment and continuous evaluation of C stocks for areas from which biomass is sourced from to qualify for EU RED targets^64^. Our results show an incremental trend in above and belowground C in live trees within procurement areas of wood pellet mills of large manufacturing capacity, and an inverse trend of C in soils in those of the US coastal southeast. Moreover, our empirical estimates can be used in greenhouse gas life-cycle assessments that to-date have relied on assumed forest management regimes within particular market scenarios to gauge the C neutrality of Trans-Atlantic wood pellet trading^65,66^.

### Competing wood fiber sectors, domestic policy, population and extreme weather

We found that an increase in the overlap between procurement areas of wood pellet mills and wood-using power plants was associated with significantly greater carbon stocks across nearly all selected C pools. As noted previously, this can be indicative of more intensive land management practices. This finding highlights that assessment of any potential associations between EU RED with timberland conditions should take into consideration other explanatory factors such as local consumption of wood fibers and domestic policies that create incentives to procure similar fibers. For instance, over the 2009–2015 period the use of wood for electricity generation in the US grew by 35% reaching 257.3 PJ in 2015^67^ with 83% generated by power plants in the eastern US.

The coefficient denoting a period of intense US bioenergy policy interventions following the Great Recession (Table 4) showed that there was a statistically significant increase in carbon stocks in live and standing-dead tree C pools. This signal might point to latent effects of other federal and state level interventions over this period that have encouraged the growth of biomass for energy, such as the US Department of Agriculture’s Biomass Crop Assistance Program^68^, and the widespread adoption of state-level best management practices for the removal of woody biomass beyond traditional timber harvesting largely implemented since 2009^69^. Prospects for the expansion of US wood pellet manufacturing are positive and some suggest that if an upward export trajectory to the EU were to continue US pellets may contribute up to 60% of annual European import demand^70^. Nonetheless, the fruition of such expectations might be contingent on air quality regulations. PM_2.5_ and PM_10_ emissions across Europe between 2005 and 2017 have reportedly increased by 11% and 7% because of growth in biomass burning, respectively, which has resulted in calls for policy action to ameliorate potential health impacts^71^.

The individual and compounded effects of EU and US renewable energy policies deserve continued examination. The US Department of Energy’s most recent outlook suggest a 21% increase in domestic electricity generation using wood by 2030^72^. The recent announcement by the US Environmental Protection Agency that in future federal regulatory actions biomass from managed forests will be treated as carbon neutral when used for energy production at stationary sources^73^ might support even greater growth in corresponding domestic wood fiber demand. Federal energy policies will continue to overlap with state-level goals such as renewable energy portfolio standards that classify wood as a renewable feedstock^74^. Continued spatially-explicit monitoring of changing timberland conditions seems to be warranted, particularly as localized demand intensifies.

Our analysis shows that population had strongly negative standardized associations with all descriptors of timberland structure and C stocks. For instance, procurement areas that hosted more inhabitants had fewer numbers of live trees and growing-stock trees likely reflecting on competing land uses. Past assessments and projections for the US eastern region consistently point to population being one of the greatest drivers of changes in timberland conditions^75,76^. Extreme drought effects showed systematic associations with several timberland conditions in the US coastal southeast. Extreme drought is likely to increase in frequency and intensity under a changing climate. Its sole and compounded effects with other anthropogenic processes should continue to be monitored explicitly.

## Conclusions

The EU, among other governments around the world^77^, are increasingly adopting policy strategies that seek the sustainable use of natural resources to support their economic development. The EU RED is a core element of the EU Bioeconomy Strategy^78^ that seeks, among other objectives, the modernization of primary energy production systems using biological resources. Environmental and sustainability issues have long been at the heart of EU policy^79,80^, yet the most recent emphasis on biological resources places even greater pressure on their utilization which could possibly counter the same environmental goals it seeks to attain^18^. The role of EU RED in the emergence of a new wood pellet industry in the US coastal southeast region, and the expansion of the industry in other areas of the eastern US in response to domestic demand and policy signals, offered the opportunity to assess related impacts on local forest resources.

Analysis of timberland conditions within wood pellet industry procurement landscapes of the eastern US showed upward and downward associations. On one hand, operation of wood pellet manufacturing in the US coastal southeast showed no significant changes in C pools of live nor standing-dead trees, and a concentration of live C in fewer growing-stock trees. Procurement areas of large-scale manufacturing facilities had higher levels of C in live-trees. Moreover, a greater overlap of wood pellet mills’ procurement areas exhibited discernible increases of selected C pools. These trends suggest that wood pellet production in the US has promoted greater growth in trees and an expansion in carbon pools in live trees—which is compliant with current EU RED biofuel trade requirements for the preservation of C stocks in biomass sourcing areas^3^. On the other hand, procurement areas of continuously operating wood pellet mills of the US coastal southeast and of large-scale capacity showed, respectively, lower carbon stocks in soils and a downward trend in the number of standing-dead trees. Understanding potential impacts of these trends on wildlife habitats and biodiversity requires additional analyses. Despite the positive association of wood pellet manufacturing with carbon in live trees, its long-term sustainability could be challenged if downward trends in basic forest structure indicators and other carbon pools were to continue.

Our findings suggest largely positive trends in timberland conditions; however, the presence of some potentially negative trends suggests that continued monitoring of localized impacts of wood pellet mill operations is important. We emphasize that monitoring must be observant of other localized conditions that can have concurrent and even greater effects than wood pellet manufacturing. Specifically, monitoring of timberland conditions within industrial procurement areas must also take into consideration population changes, expansion in wood fiber demand from other competing sectors, and extreme weather, while also controlling for overall forest ecological region conditions. Future research should examine how changes in timberland attributes affect forest biodiversity, wildlife habitat, and human well-being.

## Supplementary information

Supplementary Information

## Data Availability

The source code for our statistical learning analysis is available for download from an open source software repository located at https://gitlab.com/ashki23/rc-biomass. All data generated during this study can be reconstructed by running the source code.
