# Supplementary material for: Expansion of US wood pellet industry points to positive trends but the need for continued monitoring
Source: Sci Rep. 2020 Oct 29;10:18607. doi: 10.1038/s41598-020-75403-z (PMC7596571; doi:10.1038/s41598-020-75403-z)
Supplement: Supplementary file 1 — Supplementary Information [file 41598_2020_75403_MOESM1_ESM.docx]

**Expansion of US wood pellet industry points to positive trends but the need for continued monitoring**

**Authors:**

Francisco X. Aguilar [*Corresponding author*], Department of Forest Economics, Swedish University of Agricultural Sciences, SE-90 183, Umeå, Sweden; [francisco.aguilar@slu.se](mailto:francisco.aguilar@slu.se)

Ashkan Mirzaee, Department of Industrial and Manufacturing Systems Engineering, University of Missouri, Columbia, MO 65211, USA; [amtwc@mail.missouri.edu](mailto:amtwc@mail.missouri.edu)

Ronald G. McGarvey, Department of Industrial and Manufacturing Systems Engineering & Harry S Truman School of Public Affairs, University of Missouri, Columbia, MO 65211, USA; [mcgarveyr@missouri.edu](mailto:mcgarveyr@missouri.edu)

Stephen R. Shifley, School of Natural Resources, University of Missouri, Columbia, MO 65211, USA; [shifleys@missouri.edu](mailto:shifleys@missouri.edu)

Dallas Burtraw, Resources for the Future, Washington, DC 20036, USA; [burtraw@rff.org](mailto:burtraw@rff.org)

**Supplementary information**

**Note 1. Estimation of population within procurement areas:** Population within procurement areas was estimated by aggregating the proportional population based on the US Census county-level population within wood pellet mill procurement landscapes. The proportional population for an individual county was estimated by multiplying its population by the geographic area of the county that lies within the procurement area, and divided by the area of county^95^. This is illustrated in **Figure S3a** that shows counties within wood pellet plants’ procurement areas in West Virginia.

**Note 2. Estimation of intersections of procurement areas:** Level of intersections were calculated by aggregating the intersection of a wood pellet mill’s procurement landscape area with other pellet mills, pulp wood facilities and wood-consuming power plants’ procurement areas^95^. Values greater than 1 can occur when a pellet mill intersects with more than one overlapping facility. Since the wood pellet presence variable changes over time, corresponding intersections were calculated for all years 2005, 2008, 2011, 2014 and 2017. **Figure S3b** shows an example of intersection areas of pellet plants’ procurement areas with wood-consuming power plants’ procurement areas in Ohio.

**Note 3. Model selection:** We used Hausman test-statistics^55^ and R-squared values as a measure of goodness-of-fit to select the best fitted model specification. Model specifications included panel regressions with random and fixed effects, and spatial panel with random effects. The Hausman test examined the null hypothesis of no systematic differences between the consistent estimator (fixed effects) and efficient estimator (random effects). If the null hypothesis failed rejection, the efficient estimator is consistent. In addition to its empirical relevance to spatially-explicit data, the spatial random effects model was chosen as the best fit partly due to having the highest R-squared values and evidence of strong significance of Hausman test for most timberland attributes. Summary of these statistics are listed in **Table S1**.

**Note 4. Estimation of area-adjusted effects:** Regression coefficients can be directly interpreted as change within or between procurement areas depending on the nature of the explanatory variable. They can be more naturally interpreted if presented as mean effects per hectare within procurement areas. We note that estimation of corresponding ha-level standard errors using FIA plot data is work in progress and one must proceed with caution when estimating these within a specific landscape^96^. Per-hectare area-adjusted effects were calculated by dividing *β* coefficients by the area of procurement to find mean trees/ha/year or C/ha/year associations. The procurement area corresponded to 732,302 ha (circular area with a radius of 48 km) for the base level of manufacturing capacity (<100 thousand tons/year) and adjusted for 1,301,870 ha as the net difference in procurement area between 48 km and 80 km in radii. Interactions associated with large manufacturing capacity were divided by 2,034,172 ha (circular area of 80 km radius). Area-adjusted marginal effects are disclosed in **Table 3** and **Table 4**.

**Table S1.** Results of Hausman test-statistics and R-squared value by model specification

| **Timberland structure attributes and carbon stocks** | **Hausman test** | **Models R-squared** | | | **Spatial autoregressive coefficients** |
| --- | --- | --- | --- | --- | --- |
|  |  | **Fixed effects** | **Random effects** | **Spatial random effects** |  |
| Number of live trees (trees) | 0.000 | 0.041 | 0.463 | 0.752 | 0.451 |
| Number of growing-stock trees (trees) | 0.027 | 0.079 | 0.537 | 0.801 | 0.518 |
| Number of standing-dead trees (trees) | 0.000 | 0.158 | 0.391 | 0.616 | 0.514 |
| Carbon in live trees (tons) | 0.495 | 0.501 | 0.599 | 0.775 | 0.389 |
| Carbon in standing-dead trees (tons) | 0.151 | 0.170 | 0.402 | 0.681 | 0.361 |
| Carbon in soil (tons) | 0.996 | 0.081 | 0.440 | 0.732 | 0.428 |

**
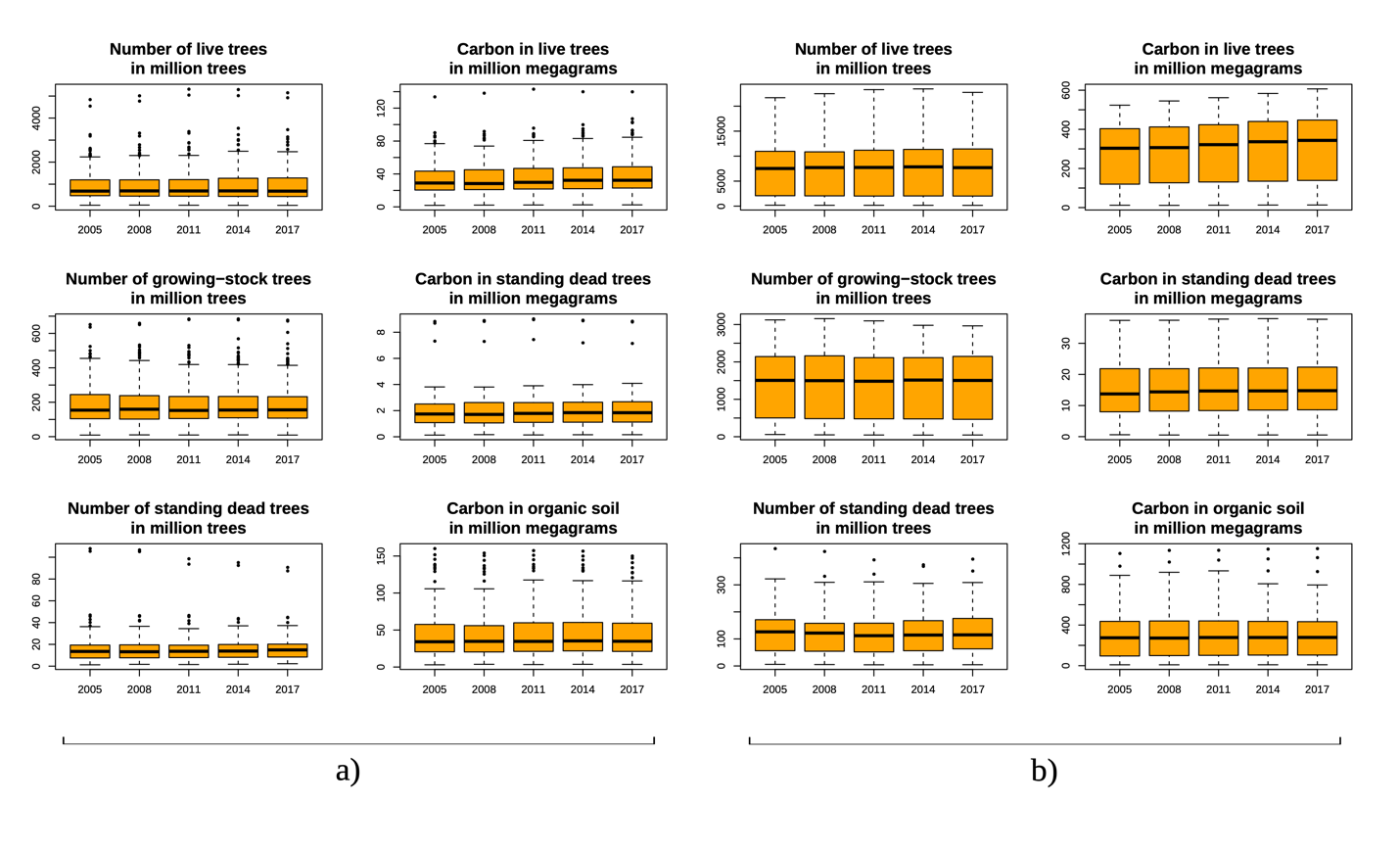
**

**Figure S1. Box-plots of annual means and ranges of attributes across (a) all 123 procurement areas, and (b) all 31 states, included in our analysis of US eastern timberland conditions.**


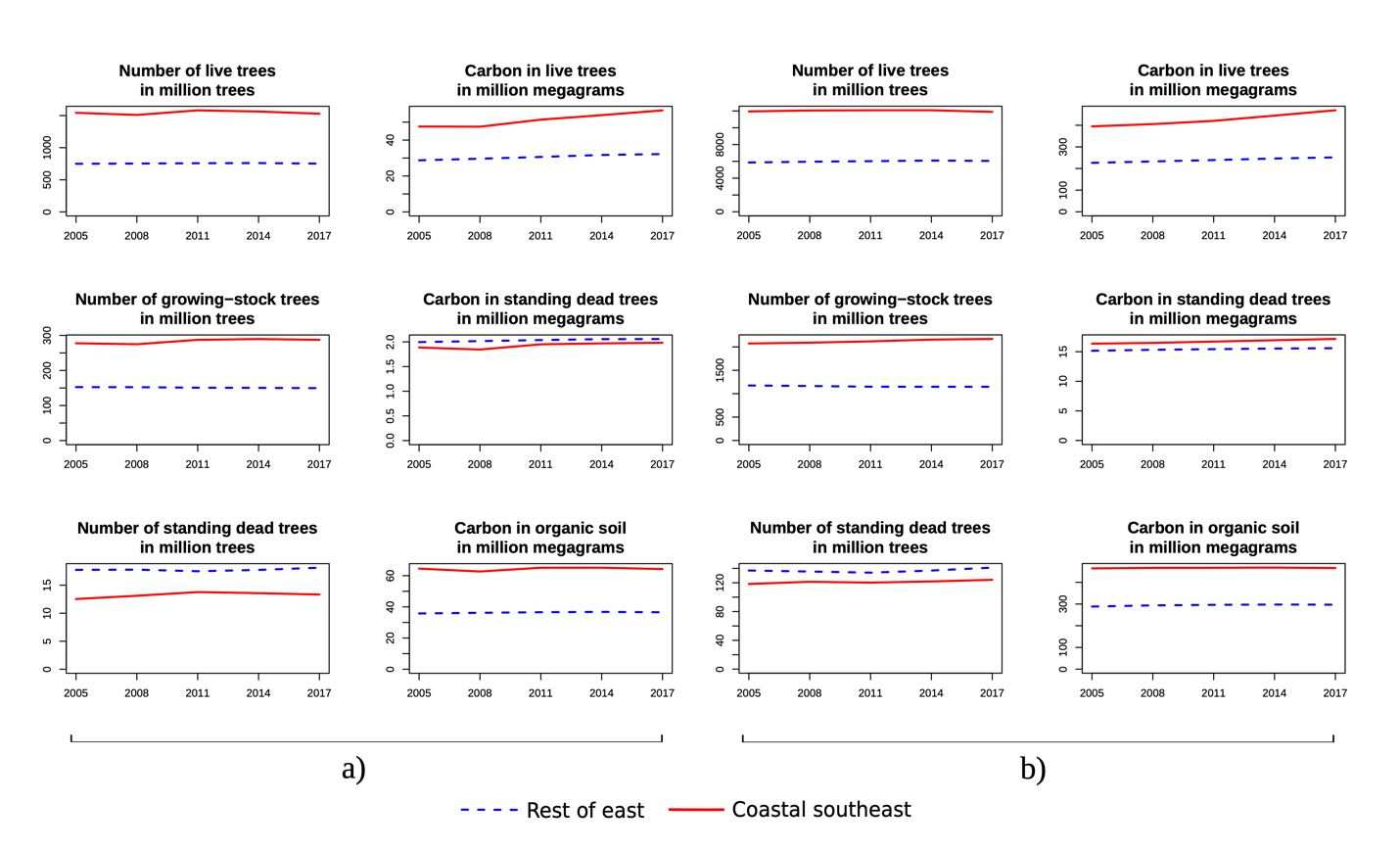


**Figure S2. Temporal** **average values of selected timberland attributes (a) within wood pellet mill procurement areas and (b) state-wide, distinguishing between US coastal southeast and the rest of eastern US.**


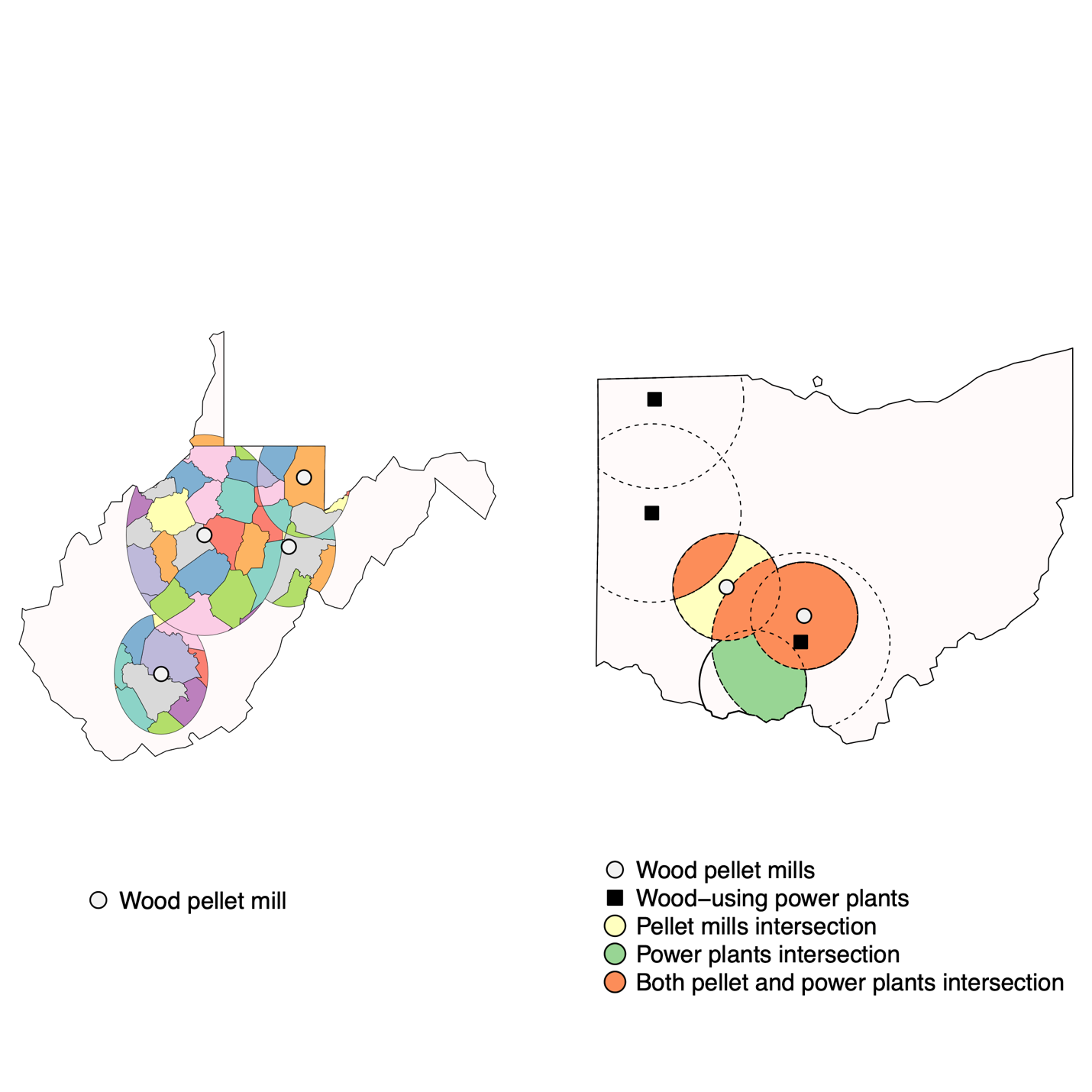


| a) | b) |
| --- | --- |

**Figure S3. Estimation of (a) population within wood pellet mill procurement areas and (b) estimation of intersections of procurement areas**. Maps generated in R using *sf* package^93,94^.
